# Supplementary material for: Light Scattering as an Easy Tool to Measure Vesicles Weight Concentration
Source: Membranes (Basel). 2020 Sep 3;10(9):222. doi: 10.3390/membranes10090222 (PMC7558410; doi:10.3390/membranes10090222)
Supplement: Supplementary file 1 [file membranes-10-00222-s001.pdf]

membranes

Supplementary Material

Light Scattering as an easy tool to measure vesicles weight concentration

Giulia Di Prima [1], Fabio Librizzi [1] and Rita Carrotta [1,*]

1 Institute of Biophysics, National Research Council, Via Ugo La Malfa 153, 90146 Palermo, Italy; giulia.diprima@virgilio.it; fabio.librizzi@pa.ibf.cnr.it

* Correspondence: rita.carrotta@cnr.it; Tel.: +39-091-6809-313

Received: date; Accepted: date; Published: 3 September 2020

Data reported as supplementary material:

- **Table 1S:** Complete calculated liposome concentrations values ± error for both POPC_LIPO and MIX_LIPO (these data are graphically reported in Figure 3).
- **From theory to practical analysis:** this section reports a step-by-step analysis for both a single extrusion of a representative sample, POPC_LIPO_50, and two independent extrusions of another representative sample, POPC_LIPO_100. It includes:
- **Figure 1S:** Static light scattered intensity measured for I and II POPC_LIPO_50 extrusions, reported as a representative example.
- **Figure 2S:** Emission elastic peaks and corresponding peak areas measured for I and II POPC_LIPO_50 extrusions, reported as a representative example.
- **Table 2S:** Experimental values, parameters and results for one POPC_LIPO_50 sample reported as a representative example.
- **Table 3S:** Calculated liposome concentrations values ± error for the two independent POPC_LIPO_100_A and POPC_LIPO_100_B preparations and their average (POPC_LIPO_100), reported as a representative example.
- **Figure 3S:** Graphical representation of data reported in Table 3S, as a representative example.

Table 1S. Calculated liposome concentrations values ± error for both POPC_LIPO and MIX_LIPO employing as the Stewart Assay technique as the novel here proposed light scattering-based method by using both a light scattering instrument (at 3 different angles) or a spectrofluorimeter.

Sample	Stewart Assay	LS-based method			
		LS instrument			Spectrofluorimeter
		LS			
		60°	90°	120°	
POPC_LIPO_50	1.02 ± 0.09	1.02 ± 0.05	1.05 ± 0.08	1.04 ± 0.08	1.04 ± 0.08
POPC_LIPO_100	1.03 ± 0.08	1.00 ± 0.02	0.99 ± 0.02	0.99 ± 0.02	0.99 ± 5·10-3
POPC_LIPO_200	1.03 ± 0.08	1.02 ± 0.07	1.00 ± 0.07	0.98 ± 0.08	0.99 ± 0.06
MIX_LIPO_50	0.92 ± 0.12	0.99 ± 0.01	0.99 ± 0.01	0.99 ± 0.02	0.99 ± 0.01
MIX_LIPO_100	1.02 ± 0.08	1.00 ± 0.07	1.00 ± 0.07	1.00 ± 0.06	1.00 ± 0.06
MIX_LIPO_200	1.05 ± 0.10	1.01 ± 8·10-3	1.00 ± 7·10-3	1.00 ± 7·10-3	1.00 ± 8·10-3

From Theory to Practical Analysis

Single extrusion

A complete procedure and data analysis for one of the prepared samples will be reported to describe the method through a representative example.

To prepare a POPC_LIPO_50 sample, 1 ml of POPC solution in chloroform (1mg/ml) was subjected to solvent casting. The obtained lipid film was rehydrated with 1 ml of bidistilled water and then subjected to FAT and extrusion cycles (lipids water concentration: 1 mg/ml).

During the extrusion procedure some crucial parameters have to be noted:

- V_T (1.400 ml): the total volume inserted into the extrusion set up. This is the sum of the lipid dispersion volume inserted for the first extrusion cycle (0.870 ml) plus the solvent volume added after the second extrusion cycle (0.530 ml).
- V_1 (0.780 ml): the volume recovered after the first extrusion cycle.
- V_2 (0.530 ml): the volume recovered after the second extrusion cycle.

These experimental values lead to calculate the following parameters:

- ΔV (0.090 ml): the unrecovered (dead) volume (which has a final C2 concentration).
- M_0 (0.870 mg): the total mass of lipid inserted into the extrusion set up. This is calculated multiplying lipids water concentration (1mg/ml) by the lipid dispersion volume inserted for the first extrusion cycle (0.870 ml).

Samples from extrusions I and II were then appropriately diluted (1:50 and 1:5 respectively) and analysed. The procedure of dilution and analysis was performed in triplicate. By employing a LS-instrument (e.g. θ=90°) two average values (Figure 1S) were obtained. For each extrusion the intensity is measured in triplicate and a value with an error is obtained for I1 and I2. Then the emission spectra were collected (Figure 2S-A) in order to calculate the areas corresponding to the elastic peak (Figure 2S-B), the area A1 corresponding to I1 and the area A2 to I2.

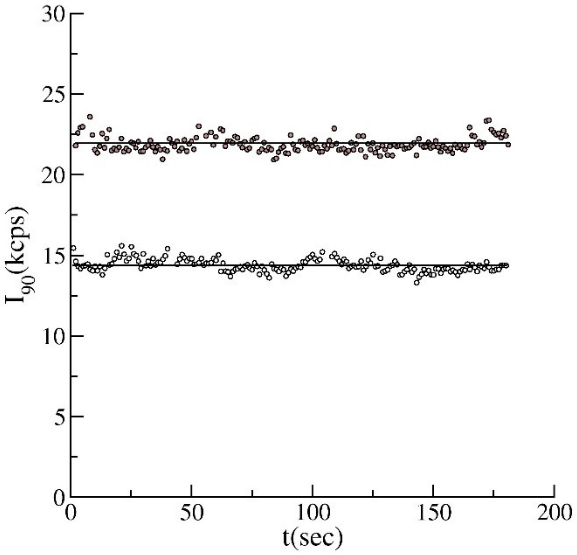

Figure 1S. Static light scattered intensity measured at θ=90° with a pin hole 100, for POPC_LIPO_50: Extrusion I (empty circle) and II (brown circle); lines represent the average value. The data have been corrected for the scattering contribution due to the solvent.

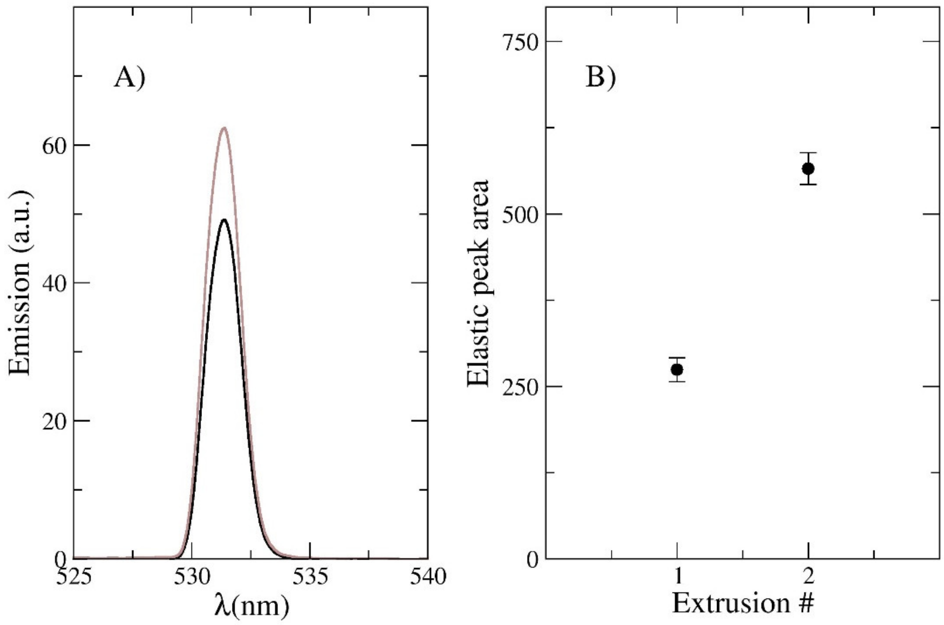

Figure 2S. A) Emission elastic peak for a representative sample of POPC_LIPO_50 (I extrusion, black line; II extrusion, brown line) and B) corresponding peak area. The data have been corrected for the emission contribution due to the solvent.

With all the collected data it is possible to calculate the liposome weight concentration by using Eq. 6 in the main text. The parameters and the obtained results of the here proposed example are reported in Table 2S.

As discussed in the Method section, the intensity and area values (average from 3 different dilutions ± SE) lead to calculate an average weight concentration ± error (determined by error propagation).

Table 2S. Experimental values, Parameters and Results for one POPC_LIPO_50 sample reported as a representative example.

**Experimental values**		**Common Parameters**		
V_1	0.780 ml	$\alpha = \frac{M_0}{V_1}$	1.115	
V_2	0.530 ml			
V_T	1.400 ml	$\upsilon = \frac{(V_T - V_1)}{V_1}$	0.795	
ΔV	0.090 ml			
M_0	0.870 mg	**Specific Parameters and Results**	**LS-instrument**	**Fluorimeter**
d_1	50	$\gamma = \frac{I_1 d_1}{I_2 d_2}$ or $\gamma = \frac{A_1 d_1}{A_2 d_2}$	6.75 ± 0.13	4.85 ± 0.61
d_2	5			
I_1	14.67 ± 0.24	$C_1 = \frac{\alpha\gamma}{\gamma + \upsilon}$	0.998 ± 0.002	0.96 ± 0.02
I_2	22.07 ± 0.25			
A_1	274 ± 33	$C_2 = \frac{\alpha}{\gamma + \upsilon}$	0.148 ± 0.003	0.20 ± 0.02
A_2	566 ± 21			

Independent extrusions

A contribution to the uncertainty in the concentration measure comes also from the film preparation and further solvation, that is from the repeatability of the sample preparation, by considering that the method assumes a known starting lipid mass, M_0. In order to evaluate this source of variability and its entity, two films were deposed for every kind of vesicle, characterized both by lipid composition and size. To discuss this point the example relative to one type of vesicle is here reported. In particular, two independent POPC_LIPO_100 samples are here considered, made from two independent lipid films, each obtained by 1 ml of POPC solution in chloroform (1mg/ml). The lipid films were hydrated with 1 ml of bidistilled water and then subjected to FAT and independent extrusion cycles with different filters of the same cut-off (lipids water concentration: 1 mg/ml). For each of the two independent preparations the fundamental extrusion parameters previously discussed were noted and the obtained samples were then appropriately diluted in order to be analysed (for each sample the dilution procedure was performed in triplicate) following the LS-based method. Results are reported in Table 3S and Figure 3S.

Table 3S. Calculated liposome concentrations values ± error for the two independent POPC_LIPO_100_A and POPC_LIPO_100_B preparations and their average (POPC_LIPO_100) for the Stewart Assay and the light scattering-based method, by using both a light scattering instrument (at 3 different angles) or a spectrofluorimeter.

		LS-based method			
**Sample**	**Stewart Assay**	**LS instrument**			**Spectrofluorimeter**
		**60°**	**LS 90°**	**120°**	
**POPC_LIPO_100_A**	1.009 ± 0.100	1.018 ± 0.006	1.011 ± 0.007	1.021 ± 0.010	0.994 ± 0.007
**POPC_LIPO_100_B**	1.058 ± 0.130	0.985 ± 0.006	0.976 ± 0.006	0.968 ± 0.006	0.989 ± 0.007
**POPC_LIPO_100**	1.033 ± 0.082	1.002 ± 0.023	0.993 ± 0.024	0.994 ± 0.037	0.991 ± 0.005

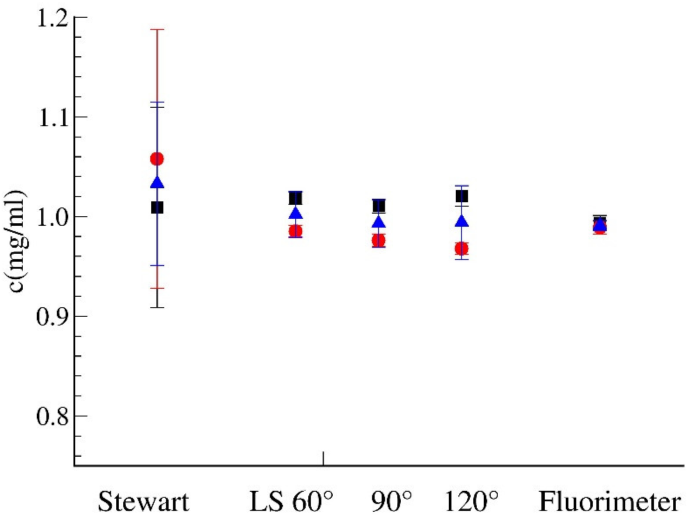

Figure 3S. Graphical representation of data reported in Table 3S for POPC_LIPO_100_A (black square), POPC_LIPO_100_B (red circle) and their average POPC_LIPO_100 (blue triangle): weight concentrations determined by the Stewart Assay and the LS-method, both using a LS instrument (60°, 90° and 120°) and a Spectrofluorimeter

As explained in the Method section, data collected from the single extrusion process were elaborated in order to obtain a unique average for each type of preparation (same composition and size). Thus, from the two average values obtained a single average weight concentration was calculated, both by Stewart Assay and LS. Two different kind of errors can be associated to this value:

- an error coming from the propagation of the experimental errors, obtained for each single preparation and reflecting the uncertainty on the measure
- an error due to the different preparation and their discrepancy, reflecting the uncertainty on the preparation procedure.

The maximum between these two was attributed to the final average value, both for Stewart Assay and LS results. It is worth to note from the values reported in Table 3S that for the Stewart assay the highest error is the "experimental" one, while for the LS based method the preparation discrepancy better represents the uncertainty on the measure.

© 2020 by the authors. Licensee MDPI, Basel, Switzerland. This article is an open access article distributed under the terms and conditions of the Creative Commons Attribution (CC BY) license (http://creativecommons.org/licenses/by/4.0/).
